# Immunoprevalence to Six Waterborne Pathogens in Beachgoers at Boquerón Beach, Puerto Rico: Application of a Microsphere-Based Salivary Antibody Multiplex Immunoassay

**DOI:** 10.3389/fpubh.2017.00084

**Published:** 2017-05-01

**Authors:** Swinburne A. J. Augustine, Kaneatra J. Simmons, Tarsha N. Eason, Clarissa L. Curioso, Shannon M. Griffin, Timothy J. Wade, Alfred Dufour, G. Shay Fout, Ann C. Grimm, Kevin H. Oshima, Elizabeth A. Sams, Mary Jean See, Larry J. Wymer

**Affiliations:** ^1^National Exposure Research Laboratory, United States Environmental Protection Agency, Cincinnati, OH, USA; ^2^Oconee Fall Line Technical College, Dublin, GA, USA; ^3^National Risk Management Research Laboratory, United States Environmental Protection Agency, Cincinnati, OH, USA; ^4^Oak Ridge Institute for Science and Education, Oak Ridge, TN, USA; ^5^National Health and Environmental Effects Laboratory, Research Triangle Park, NC, USA

**Keywords:** multiplex, immunoassay, salivary antibody, saliva, exposure, bead-based, immunoprevalence, coimmunopositivity

## Abstract

Waterborne infectious diseases are a major public health concern worldwide. Few methods have been established that are capable of measuring human exposure to multiple waterborne pathogens simultaneously using non-invasive samples such as saliva. Most current methods measure exposure to only one pathogen at a time, require large volumes of individual samples collected using invasive procedures, and are very labor intensive. In this article, we applied a multiplex bead-based immunoassay capable of measuring IgG antibody responses to six waterborne pathogens simultaneously in human saliva to estimate immunoprevalence in beachgoers at Boquerón Beach, Puerto Rico. Further, we present approaches for determining cutoff points to assess immunoprevalence to the pathogens in the assay. For the six pathogens studied, our results show that IgG antibodies against antigens from noroviruses GI.I and GII.4 were more prevalent (60 and 51.6%, respectively) than *Helicobacter pylori* (21.4%), hepatitis A virus (20.2%), *Campylobacter jejuni* (8.7%), and *Toxoplasma gondii* (8%) in the saliva of the study participants. The salivary antibody multiplex immunoassay can be used to examine immunoprevalence of specific pathogens in human populations.

## Introduction

Acute gastrointestinal illness (AGI) has long been associated with swimming in fecally contaminated waters (1–12). Epidemiological surveys and enzyme-linked immunosorbent assays are often used to ascertain the cause of these illnesses. These approaches are time consuming, costly, and suffer from challenges such as selection bias and patient recollection of symptoms (13, 14). Further, AGIs may be caused by many different pathogens, including a number of viruses, bacteria, and protozoa.

Multiplexed immunoassays have been primarily used to assess antibodies in serum; however, oral fluid represents a promising alternative to serum in studies to determine the presence and incidence of certain infections (15–26). Saliva is simple and safe to collect, well tolerated by children, non-invasive, inexpensive compared to serum, and importantly can usually be self-collected. Antibodies in saliva have been detected against bacteria (27–32), protozoa (33–36), and viruses (20, 37–44). The combination of saliva samples and a multiplex assay provides a powerful tool that could enhance health outcome assessment and measurement for certain types of epidemiological studies (17, 19, 35, 45, 46).

Several potentially waterborne pathogens, including *Campylobacter jejuni, Helicobacter pylori, Toxoplasma gondii*, hepatitis A virus (HAV), and noroviruses, were selected for this study based on their relative importance for public health and availability of immunogenic proteins. These organisms may have multiple routes of transmission, but all have been implicated as possible sources of waterborne disease. Specifically, *C. jejuni* is a common cause of acute bacterial gastrointestinal illness, often associated with foodborne disease, primarily from poultry and raw milk (47). However, outbreaks of campylobacteriosis due to contamination of drinking (47–49) and recreational (50, 51) waters have been reported. *H. pylori* is a bacterium uniquely adapted to chronically colonize the human stomach, which causes transient acute dyspeptic symptoms following initial colonization (52), but chronic infection can cause chronic gastritis, gastric or duodenal ulcers, and gastric cancer (53). Although most *H. pylori* infections are believed to be acquired *via* person-to-person transmission, waterborne transmission is also possible (54) due to the ability of *Helicobacter* to contaminate water supplies (55) and survive in distribution system biofilms (56). *T. gondii* is a parasite of felines, which also infects a wide variety of intermediate hosts, including livestock and humans (57). Infections are benign in most immunocompetent individuals (57), but *Toxoplasma* infection in a previously uninfected pregnant woman can cause miscarriage or neurological damage to the fetus (58). The predominant route of toxoplasmosis infection in humans is ingestion of undercooked meat; however, disinfection-resistant oocysts excreted by cats can also cause waterborne outbreaks (59–61). Furthermore, epidemiological associations have been reported between well water use and *T. gondii* antibody prevalence (62–64). HAV is an RNA virus that causes a highly contagious liver infection. It is typically transmitted by the fecal–oral route, either through consumption of contaminated food or water or *via* person-to-person contact (65). It was previously demonstrated that areas with inadequate water supply and poor wastewater facilities and hygienic conditions generally have high HAV prevalence (65, 66). HAV outbreaks have also been associated with drinking (67) and recreational water exposures (68). Finally, noroviruses are a diverse group of RNA viruses, which are a major cause of acute gastroenteritis worldwide. Transmission of noroviruses may occur *via* ingestion of contaminated food or water, exposure to contaminated fomites, and person-to-person contact. Noroviruses can contaminate surface waters (69) and cause outbreaks associated with chlorinated water supplies (70, 71) and untreated ground water (67, 72). Outbreaks have also been associated with recreational swimming exposures in lakes (50, 73, 74).

To study human exposure to these potentially waterborne pathogens, we previously developed a Luminex xMAP™ bead-based, salivary IgG antibody multiplex immunoassay to measure antibodies to *C. jejuni, H. pylori, T. gondii*, HAV, and noroviruses GI.1 and GII.4 (14, 19). Assay parameters such as antigen concentration and coupling, cross-reactivity, sensitivity, and specificity, as well as anti-human detection antibody and reporter concentrations, were optimized and tested using both characterized human plasma and saliva samples (14, 19).

Here, we used our optimized multiplex immunoassay (14) consisting of recombinant proteins, whole cells, and cell lysates to MicroPlex™ beads to indirectly capture human antibodies in saliva samples collected from visitors to Boquerón Beach, Puerto Rico. Methods were presented to examine potential patterns and estimate exposure in the population. The approaches described here allowed us to determine the prevalence of waterborne infections by using IgG antibodies as biomarkers of exposure in non-invasively collected human saliva samples. The goal of this study was to describe the method and overall prevalence of exposure. Subsequent manuscripts will address exposure routes and rates of new infections (immunoconversions).

## Materials and Methods

### Reagents

Bead sets were obtained from Luminex Corp. (Austin, TX, USA) at a concentration of 12.5 × 10^6^ beads/ml each. Biotin-labeled affinity purified goat anti-human IgG (λ) secondary detection antibody was obtained from KPL (Gaithersburg, MD, USA). Antigens were purchased (as shown in Table 1) and coupled to the beads in accordance with the optimized multiplex immunoassay presented in the study by Augustine et al. (14). The optimized conditions were applied to the antigens multiplexed in this study.

**Table 1:** **Antigens, sources, and coupling concentrations used in the multiplex immunoassay**.

Organism	Antigen	Source	Amt. of Ag coupled (μg)
*Campylobacter jejuni*	Heat-killed whole bacterial cells	KPL	50
*Helicobacter pylori*	Bacterial cell lysate	Meridian	25
*Toxoplasma gondii*	Recombinant p30 (SAG1)	Meridian	25
Hepatitis A virus	Cell culture concentrate	Meridian	100
Norovirus GI.1	P-particle	Xi Jiang^a^	5
Norovirus GII.4	P-particle	Xi Jiang^a^	5

*^a^Cincinnati Children’s Hospital Medical Center*.

### Antigen Coupling and Confirmation Using Animal-Derived Antibodies

The Luminex beads were activated and coupled, as previously described (14, 16, 19). Coupling confirmation was performed using serial dilutions of commercially available, animal-derived primary capture antibodies specific to each antigen to ensure that the beads were sufficiently coupled and that the dynamic range of the assay could be defined (14). Briefly, a working bead mixture was prepared by diluting the coupled bead stocks to a final concentration of 100 beads/μl of each unique bead set in phosphate-buffered saline containing 1% bovine serum albumin (PBS-1% BSA). Twofold serial dilutions of anti-species IgG primary antibody were prepared per the manufacturer’s recommendations. Then 5 × 10^3^ beads from each bead set were added to each well of a prewet filter plate. An equal volume of the serially diluted species-specific antibody was added to the beads, mixed gently, covered, and allowed to incubate in the dark at room temperature for 30 min at 500 rpm on a VWR™ microplate shaker (Radnor, PA, USA).

The supernatant was removed by vacuum, the wells were washed twice with 100 µl of PBS pH 7.4 containing 0.05% Tween 20 (PBS-T) (Sigma, St. Louis, MO, USA), and excess buffer was removed by vacuum. The beads were gently resuspended in PBS-1% BSA buffer, and 0.8 µg of biotinylated anti-species IgG secondary detection antibody was added to each well. The filter plates were covered and allowed to incubate in the dark at room temperature for 30 min on a plate shaker. After incubation, the wells were washed twice with 100 µl of PBS-T, and excess buffer was removed as mentioned earlier. Finally, 1.2 µg of streptavidin-R-phycoerythrin was added to each well, mixed gently, incubated for 30 min, and washed twice as mentioned earlier. Excess buffer was removed by vacuum, the beads were resuspended in 100 µl of PBS-1% BSA, and the plate was analyzed on a Luminex 100 analyzer (Luminex Corporation, Austin, TX, USA).

### Saliva Collection, Processing, and Analysis

Approval was obtained from the institutional review board (# 08-1844, University of North Carolina, Chapel Hill, NC, USA) for the collection of saliva samples from beachgoers at Boquerón Beach, Puerto Rico, as a part of the USEPA National Epidemiological and Environmental Assessment of Recreational Water Study (75). Households were offered enrollment on a first-come, first-served basis each day until a goal of 100 was reached. Study subjects (*n* = 2,091) provided informed consent and were instructed on the use of the Oracol™ saliva collection device (Malvern Medical Developments, Worcester, UK). Briefly, the Oracol™ sampler was rubbed against the gingival crevices of the oral mucosa (between the gums and teeth) to absorb saliva. Infants younger than 1 year were not included due to the possible presence of maternal antibodies and high rates of non-waterborne infections. Individuals who reported dental or other illnesses at the time of the initial collection were also excluded.

The baseline samples were collected on the beach by trained study staff members. Upon receipt, samples were cataloged using FreezerWorks™ software (Dataworks Development, Inc., Mountlake Terrace, WA, USA) and stored at −80°C until tested. Oracol™ saliva collection devices were thawed at room temperature and centrifuged at 491 × *g*, 10°C, for 5 min to recover the saliva off the collection sponge, followed by another centrifugation at 1,363 × *g*, 10°C, for an additional 5 min to pellet debris from the saliva. After centrifugation, the saliva was aspirated from the tubes and transferred to 1.5-ml microcentrifuge tubes. The saliva containing tubes were centrifuged at 1,500 × *g* for 3 min, and the supernatant was transferred to a clean 1.5-ml microcentrifuge tube and either used immediately for analysis or stored at −80°C.

Before analysis, samples were diluted 1:4 in PBS-1% BSA in a 96-well round bottom plate (Corning™, Corning, NY, USA) and gently mixed. MultiScreen BV 96-well filter plates (Millipore, Billerica, MA, USA) were prewetted with 100 µl of PBS-1% BSA buffer, and excess buffer was removed by vacuum. Then 5 × 10^3^ beads from each bead set and an equal volume of diluted saliva were loaded onto each well of the filter plates resulting in a final dilution of 1:8 for a total volume of 100 µl per well. The loaded filter plates were processed, as previously described (16, 19), and reporter fluorescence was measured using a Luminex 100 analyzer and expressed as median fluorescence intensity (MFI) of at least 100 beads per bead set.

### Assay Controls, Cross-Reactivity, and Signal-to-Noise Ratio (SNR)

One set of Luminex beads was used as a no-antigen (uncoupled) control to assess the extent of non-specific binding and sample-to-sample variability. These beads were treated identically to antigen-conjugated beads with the exception that control beads were not incubated with any antigen during the coupling step. As with the antigen-coupled beads, the control beads were blocked with BSA, a protein that is a key reagent in the buffers used to perform the assay.

Of the 2,091 saliva samples obtained from study participants, 13 samples were observed to react with the control beads. These samples, likely outliers in the 99th percentile of the control beads, were subsequently removed from analyses due to the potential for contamination of the saliva by serum from bleeding gums. The remaining samples (*n* = 2,078) were used in the assessment. Cutoff points for immunopositivity were derived from the MFI values of these uncoupled beads (control). In addition, background fluorescence and cross-reactivity of antigen-coupled beads to secondary detection antibodies and/or reporter were evaluated by adding PBS-1% BSA buffer to control wells instead of saliva. The MFI values from the background controls (antigen-coupled beads with PBS-1% BSA) were subtracted from the MFI obtained from every antigen-coupled bead set for each saliva sample.

Additional tests of cross-reactivity were performed with animal-derived primary detection antibodies, including monoplex (one antigen-coupled bead set with its specific primary antibody and controls), duplex (two antigen-coupled bead sets and one primary antibody and controls), and multiplex (six antigen-coupled bead sets and uncoupled control beads with one primary antibody). Bead coupling and confirmation, cross-reactivity, and method validation using characterized plasma samples were performed, as previously described (14).

As a surrogate measure of assay sensitivity, a SNR was calculated by dividing the MFI of the specific antigen signals by the MFI of the uncoupled control beads for each sample (14, 76). We computed a SNR for each reading as a ratio of the MFI value for sample *i* when tested for antibodies to antigen *j*(*R_ij_*) and the MFI value of the uncoupled control for the sample (*C_i_*):

(1) $${\text{SNR}}_{\mathrm{ij}}=\frac{R_{\mathrm{ij}}}{C_{i}}.$$

### Approaches to Determine Background Cutoff Points to Investigate Antibody Prevalence

We explored two approaches to establish a means of estimating immunoprevalence to all targets in the Luminex multiplex assay in the absence of information regarding true infection status of the participants. To help determine immunoprevalence, researchers have used a variety of methods including three times the mean (77) and mean plus 3 times the standard deviation (SD) of the control beads (16, 78). Cutoff point criteria 1 (CC1) is defined as the mean plus 3 SDs (μ + 3σ) of the control beads. As a basic test for normality, histograms were generated, which reflected positive skewness in the salivary antibody responses observed in this study population. Cutoff point criteria 2 (CC2) was established to account for this non-normality and is defined as the antilog of the mean plus 3 SDs of the log_10_-transformed data [10^mean(^*^h^*^)+3SD(^*^h^*^)^, where *h* = log_10_(control MFI)]. All data analysis was performed using Microsoft Excel 2013 and MATLAB Release 2016a.

## Results

### Study Population

Since we were interested in only assessing the immunoprevalence for the antigens in the multiplex for this study, IgG levels against the six antigens in the Luminex multiplex immunoassay were measured in baseline saliva samples collected from 2,078 consenting individuals at Boquerón Beach, Puerto Rico.

### Summary of Antibody Data Analysis

**Figure 1:**
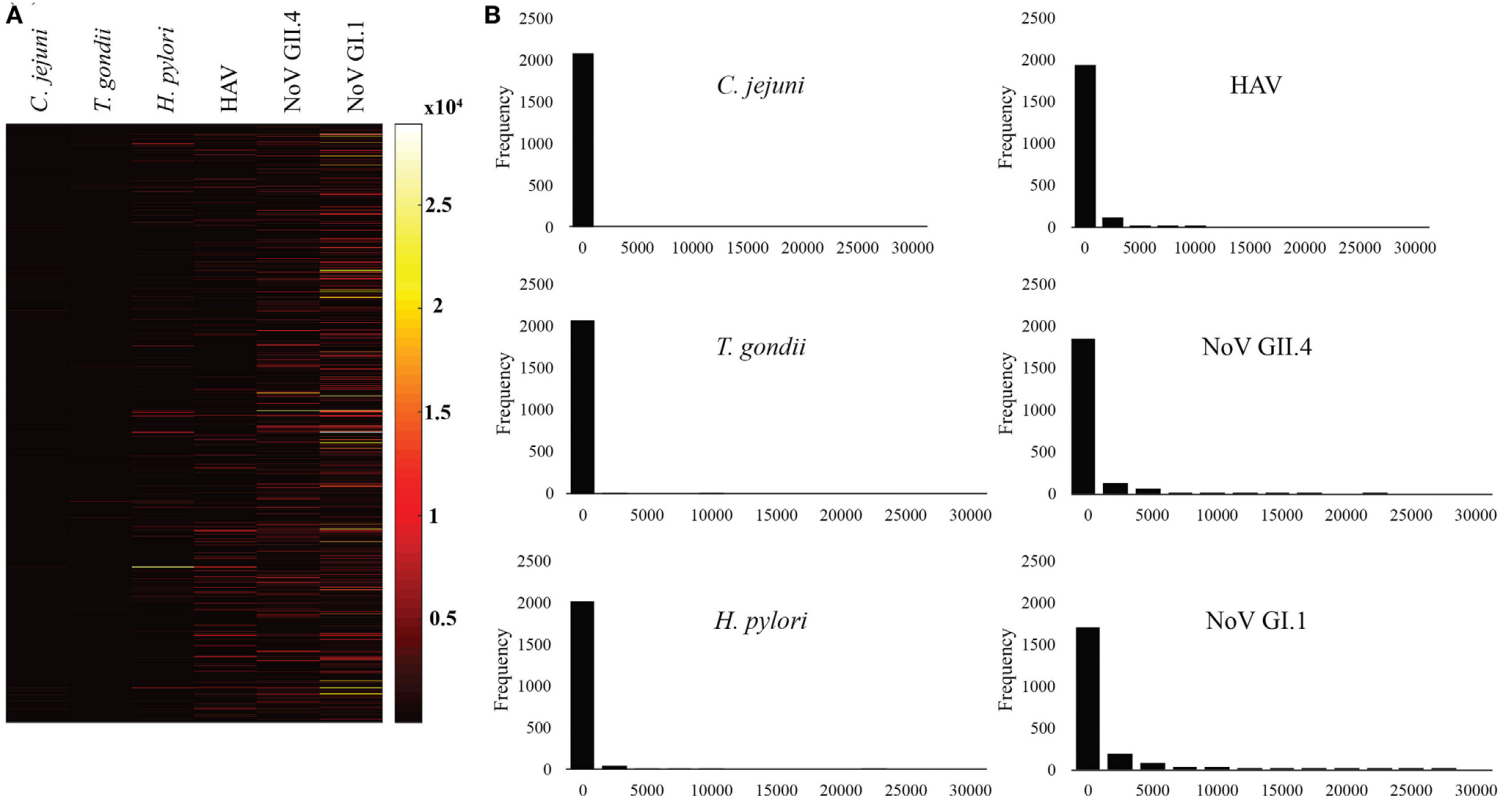
**Median fluorescence intensity (MFI) profile of study participants**. **(A)** Heat map visualization of study participants’ antibody profiles to six waterborne pathogens provides insight on possible exposure patterns. Each row presents the MFI values of the target pathogens for the individual saliva sample collected from beachgoers. **(B)** Histograms showing the non-normal distribution of target pathogen MFIs for the study participants.

**Figure 2:**
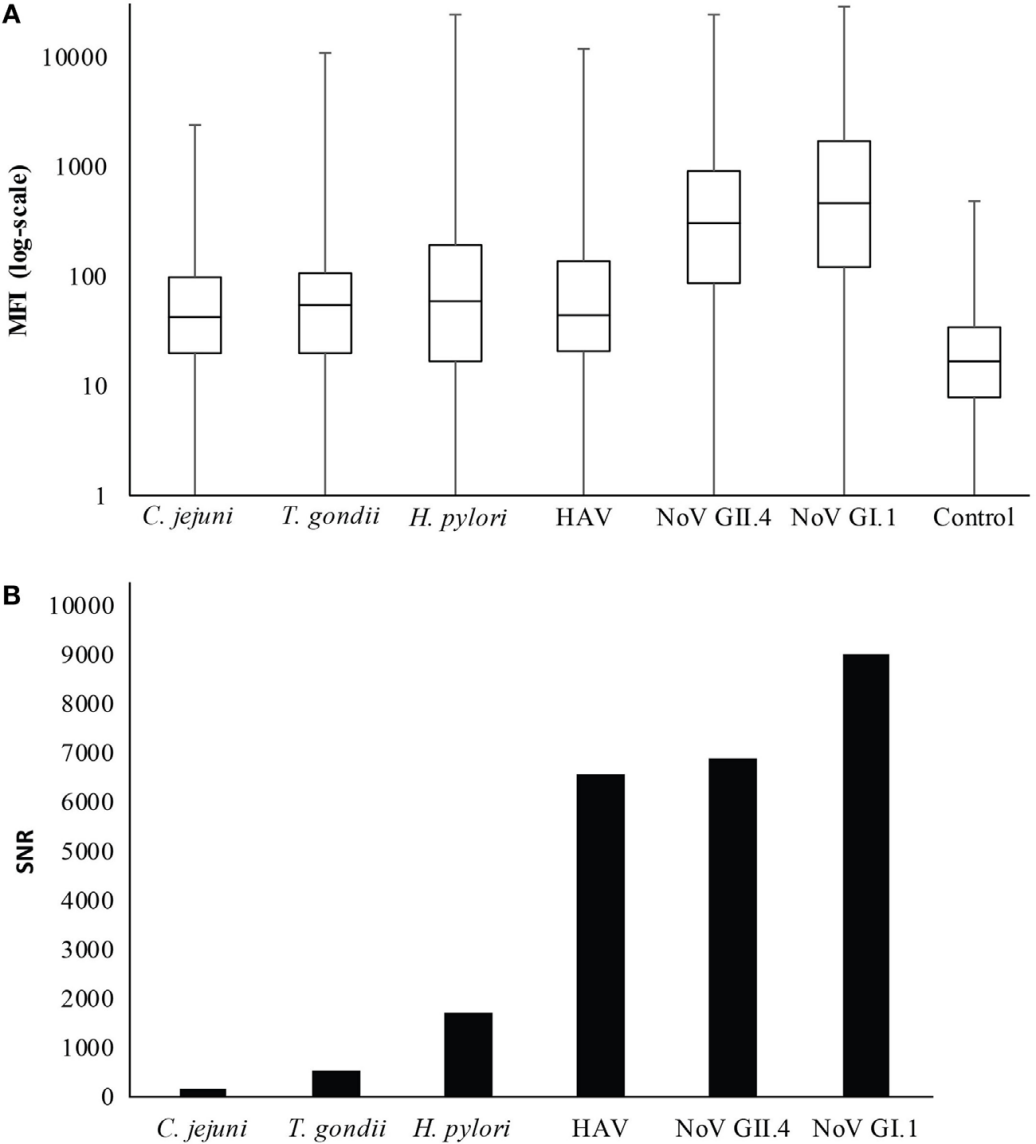
**Distribution of median fluorescence intensity (MFI) results**. **(A)** Boxplot of MFI results in log scale. The whiskers represent the minimum and maximum values. **(B)** Maximum signal-to-noise ratio (SNR) for each antigen in the multiplex immunoassay.

Figures 1 and 2 summarize the study participants’ antibody profiles to the six waterborne pathogens. In Figure 1A, each row of the heat map represents the MFI values of the target pathogens for an individual saliva sample and provides insight on potential exposure patterns. The MFI values for each target is right skewed, reflecting a non-normal distribution (Shapiro–Wilk test *p*-values <0.05) (Figure 1B). Figure 2A shows that each antigen is characterized by a broad range of MFI values showing noroviruses with the largest median MFIs. The maximum SNRs for each individual antigen in the multiplex assay ranged from 145:1 (*C. jejuni*) to 9,004:1 for norovirus GI.1 (Figure 2B). Here, we define immunoprevalence as a baseline sample (*n* = 2,078) with a MFI reading greater than or equal to the cutoff point established.

### Establishing Cutoff Points and Assessing Immunoprevalence

**Figure 3:**
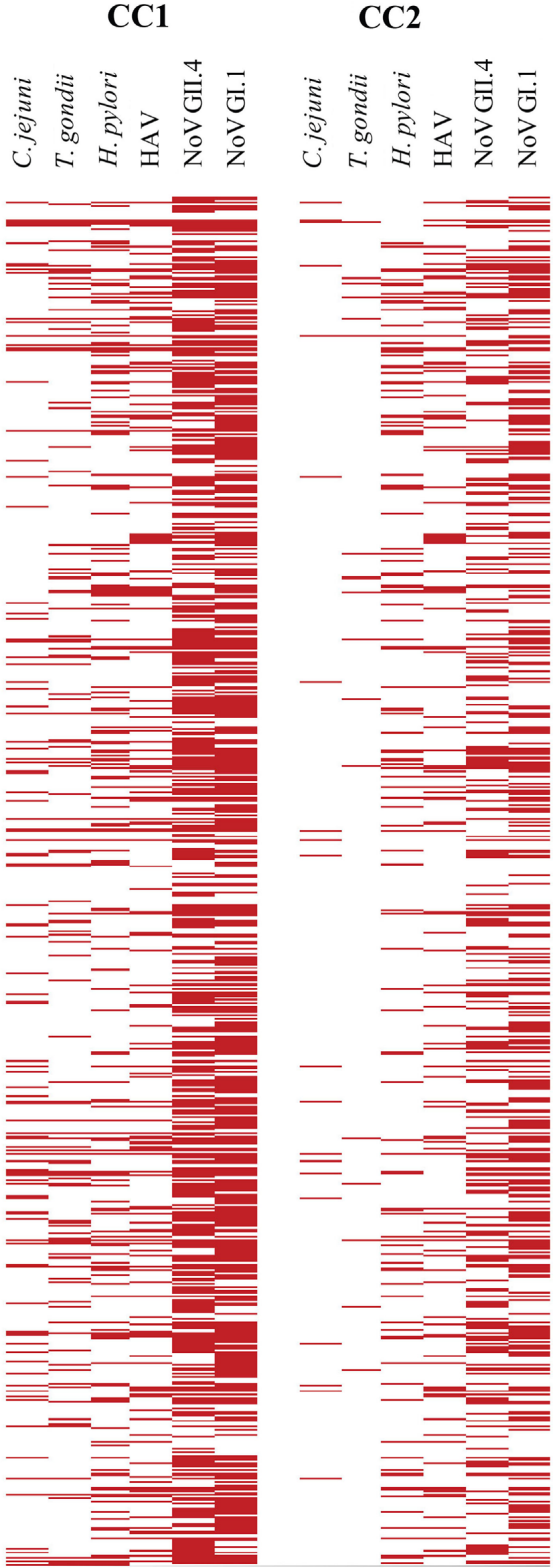
**Visualization of cutoff point ratios**. The left panel shows the positive samples based on cutoff point criteria 1 (CC1) [150 median fluorescence intensity (MFI)], and the right panel indicates positive samples based on cutoff point criteria 2 (CC2) (505 MFI). Ratios over 1 (MFI ≥ cutoff point) are red in the plots. MFIs below cutoff point are white.

Table 2 provides the cutoff points and immunoprevalence results for CC1 (87.6%) and CC2 (67.7%), and Figure 3 shows the corresponding visualization. Table 3 (upper panel) provides a breakdown of the immunoprevalence to specific pathogens with evidence of the greatest amount of exposure to noroviruses GI.1 (48.6–71.2%) and GII.4 (37.6–65.7%) followed by *H. pylori* (14–28.7%), HAV (16.2–24.3%), *T. gondii* (2–14%), and *C. jejuni* (2.3–15.2%).

**Table 2:** **Methods for determining cutoff points to estimate immunoprevalence in saliva samples**.

Cutoff point criteria		Cutoff point	# (%)
Cutoff point criteria 1 (CC1)	Mean + 3 SD	150	1,821 (87.6)
Cutoff point criteria 2 (CC2)	10^mean(^*^h^*^)+3SD(^*^h^*^)^	505	1,406 (67.7)

*CC1: mean plus 3 SDs [CC1 = 150 median fluorescence intensity (MFI)] and CC2: 10^mean(^^h^^)+3SD(^^h^^)^ (CC2 = 505 MFI). h = log_10_(MFI of control beads)*.

*# (%) represents the number (percentage) of immunopositive samples based on the cutoff used*.

**Table 3:** **Immunoprevalence rates to specific (upper panel) and multiple pathogens simultaneously (lower panel)**.

To specific pathogens, *n* (%)		
Pathogens	Cutoff point criteria 1 (CC1)	Cutoff point criteria 2 (CC2)
*Campylobacter jejuni*	315 (15.2)	47 (2.3)
*Toxoplasma gondii*	291 (14.0)	41 (2.0)
*Helicobacter pylori*	597 (28.7)	291 (14.0)
Hepatitis A virus	504 (24.3)	336 (16.2)
NoV GII.4	1,365 (65.7)	781 (37.6)
NoV GI.1	1,479 (71.2)	1,009 (48.6)

**To *N* pathogens simultaneously, *n* (%)**		
***N***	**CC1**	**CC2**

0	257 (12.4)	672 (32.3)
1	435 (20.9)	638 (30.7)
2	622 (29.9)	510 (24.5)
3	412 (19.8)	195 (9.4)
4	201 (9.7)	53 (2.6)
5	74 (3.6)	10 (0.5)
6	77 (3.7)	0 (0.0)

**Summary**		
	**CC1**	**CC2**

None	257 (12.4)	672 (32.3)
Single (*N* = 1)	435 (20.9)	638 (30.7)
Multiple (*N* ≥ 2)	1,386 (66.7)	768 (37.0)

### Defining and Measuring Coimmunopositivity

Based on our cutoff points, 1,386 (CC1) and 768 (CC2) samples showed salivary antibody responses to multiple (*N*) antigens (Table 3, lower panel). This prompted us to investigate whether this was a result of cross-reactivity or true coimmunopositivity. The MFI values of the complete baseline data set (*n* = 2,078) were used to calculate coimmunopositivity and assess correlation for all the multiplexed antigens in the immunoassay. Table 4 shows the numbers and percentages of pairwise analyses of the multiplexed antigens using both the less stringent cutoff point criteria (CC1, upper panel) and a more conservative cutoff point criteria (CC2, lower panel). The Spearman rank-order correlation coefficient (rho) assessing the linear relationship between the MFI of the antigens in the multiplex ranged from 0.20 for *H. pylori*/norovirus GII.4 to 0.55 for *C. jejuni*/*H. pylori* (*p*-value < 0.05 for each pairing), indicating a statistically significant, weak to moderate positive correlation.

**Table 4:** **Numbers and percentages of coimmunopositive samples observed in baseline samples based on cutoff point**.

Cutoff point criteria 1 (CC1)						
	*Campylobacter jejuni*	*Toxoplasma gondii*	*Helicobacter pylori*	Hepatitis A virus (HAV)	Nov GII.4	Nov GI.1
*C. jejuni*	X	130 (6.26)	176 (8.47)	143 (6.88)	260 (12.51)	287 (13.81)
*T. gondii*		X	163 (7.84)	135 (6.50)	233 (11.21)	264 (12.70)
*H. pylori*			X	286 (13.76)	445 (21.41)	544 (26.18)
HAV				X	360 (17.32)	448 (21.56)
Nov GII.4					X	1,115 (53.66)
Nov GI.1						X

**Cutoff point criteria 2 (CC2)**						
	***C. jejuni***	***T. gondii***	***H. pylori***	**HAV**	**Nov GII.4**	**Nov GI.1**

*C. jejuni*	X	4 (0.19)	15 (0.72)	17 (0.82)	30 (1.44)	41 (1.97)
*T. gondii*		X	15 (0.72)	11 (0.53)	25 (1.20)	31 (1.49)
*H. pylori*			X	98 (4.72)	123 (5.92)	211 (10.15)
HAV				X	135 (6.50)	241 (11.60)
Nov GII.4					X	519 (24.98)
Nov GI.1						X

*CC1 (upper panel) and CC2 (lower panel) results indicate the percent of samples that are simultaneously immunopositive to the antigen pairs based on the cutoff points (MFI ≥ cutoff point)*.

## Discussion

This is the first multiplex immunoassay study aimed at describing the immunoprevalence of circulating antibodies to six waterborne pathogens in beachgoers in Puerto Rico. The identification of waterborne pathogen immunoprevalence is a necessary and highly important first step in assessing and managing recreational water-associated AGI risks. Analysis of the initial saliva samples for antibodies to these six pathogens revealed that more than two-thirds of the beachgoers were previously infected by at least one of the pathogens studied. On average, the results indicated that more than three-fifths of the beachgoers had evidence of prior exposure to noroviruses, while over a fifth were previously exposed to *H. pylori* and HAV. Antibodies against *T. gondii* and *C. jejuni* were less common. These results are relatively consistent with previously published data that show the prevalence of infections from noroviruses GII.4 and GI.1 at >80% worldwide (79). *H. pylori* prevalence in developed countries ranged from 30 to 40% and 80 to 90% in the developing world (80). The seroprevalence of HAV in the United States was reportedly 34.9% overall (81), while *C. jejuni* and *T. gondii* were 15–30% and 7–22.5% prevalent, respectively (63, 82, 83). Taken together, the initial sample results suggest that these individuals had a historical exposure to the pathogens being studied. Future studies will investigate immunoconversions related to swimming-associated exposures.

Of the beachgoers who were previously exposed, many (37–67%) were found to have evidence of detectable antibodies to two or more antigens from our samples. This finding is consistent with previous studies of AGI performed in multiplex (84–87). These observations demonstrate the potential for multiplex assays to identify multiple infections and may be applied either crosssectionally or longitudinally to provide insight into the epidemiology of these conditions, as well as understanding the role of potential risk factors.

Selection of the definition to determine the cutoff points and immunoprevalence may depend on the study question being answered. For example, a less stringent criterion may be acceptable for a simple screening of samples requiring further clinical diagnostic testing. However, for studies where the immunological status of the subjects is not known or the data are not normally distributed, as is the case here, the more stringent definition is strongly preferred to reduce false positives. Although the more conservative cutoff point may reduce false positives, it may have the opposite effect of increasing false negatives because we do not know the immunological status of the study participants.

In a previous study (14), we described the performance of the multiplex immunoassay to measure IgG antibodies using characterized human plasma samples against the antigens that we have used here. In that study, we described, in detail, the process used to address cross-reactivity using characterized (diagnostically positive and negative) plasma samples. Briefly, multiplexed antigens that showed cross-reactivity >10% to antigen-specific, animal-derived antibodies were removed from the immunoassay. A clear case of cross-reactivity was observed with the rabbit anti-*Giardia duodenalis* antibody. That particular antibody bound to every antigen in the multiplex immunoassay at MFI levels that ranged from 59 to 180% (14) of the target antigen. Furthermore, we found the sensitivity of the assay to be ~92% based on the number of characterized samples that were correctly identified as being either positive or negative.

We have also reported on the development of multiplex immunoassays using saliva samples (16, 19). We observed that a 1:8 dilution of the saliva, together with the addition of 0.05% Tween 20 to the wash buffer, resulted in decreased non-specific binding. The optimal concentrations for each antigen used in the multiplex are shown in Table 1. Two approaches to determine cutoff points to define immunopositivity were considered for interpreting results of the multiplexed immunoassay to measure salivary IgG antibodies. The purpose was to examine the effects of the two options on the interpretation of the results and to make recommendations on which cutoff point might be the most appropriate for measuring immunoprevalence in a population. The cutoff point will also help inform which approach is most useful for the assessment of incident infections and health effects studies for waterborne infections.

We recognize that there are several limitations to our study. First, the possibility of promiscuous antibody binding with other antigens cannot be totally eliminated due to cross-reactivity (binding to similar or overlapping ligands) or multispecificity (binding of distinctly different ligands or different conformations of the same antibody) (88–91). The data presented in Tables 3 and 4 provide some evidence that for each pair of antigens, there is a statistically significant weak to moderate positive correlation (*p*-value < 0.05), suggesting either the possibility that these infections were occurring concomitantly as a feature of the study population or some level of cross-reactivity. The percentages of pairwise binding obtained using CC1 appear to show more coimmunopositivity, but those numbers are drastically reduced when the more stringent CC2 is employed. We explored using PVX (polyvinylalcohol + polyvinylpyrrolidone + 0.05% Tween™ 20) buffer to reduce non-specific binding (92–94) and found that PVX buffer had a dramatic effect on reducing non-specific binding in plasma, which was not observed in saliva (data not shown). These results agree with observations made in a previous study (20).

Another limitation of the study is that antibody levels in saliva samples are typically lower than levels observed in sera or plasma. In this study, we found that the maximum SNR ranged from 145:1 to 9004:1, indicating a wide dynamic range for antigens in the assay. Some individuals with low specific serum IgG will have even lower saliva IgG concentrations, and therefore, these individuals may not be considered when reporting immunoprevalence. For example, in studies where vaccination coverage rates were examined, it was noted that a number of participants fell within a “gray zone” (above the negative cutoff point but below the positive cutoff point) using saliva (39). This was attributed to differences in antibody levels between individuals with wild-type virus or vaccine-induced immunity. It was found that sensitivity of the oral fluid method increased as antibody titers in serum increased. The implication of this finding is that immunoprevalence may be underestimated by oral fluid IgG detection because the assay may not have sufficient sensitivity when the serum antibody titer range is low.

The largest concentration of IgG found in the oral cavity gains access to this region by passive diffusion from the blood stream using the crevicular epithelium of the gingiva, while the remaining concentration of IgG is due to the presence of plasma cells in the crevicular epithelium (95). To account for the lower concentrations of IgG in saliva, especially compared to IgA, we employed the Oracol oral fluid sampler, which is designed to collect crevicular fluid enriched with serum antibodies (21). Our study focused on measuring systemic rather than mucosal immune responses in saliva, exclusively of the IgG isotype. This is, in part, because salivary IgA levels are especially influenced by a donor’s age, secretory flow rate, acute and chronic stresses, and other methodological issues (18). In addition, our previous research indicated that IgA from saliva samples produced weaker responses compared to IgG in saliva (19, 20).

In spite of these limitations, the utility of the multiplex saliva assay is that it can be used for population-based immunoprevalence studies, which can be further exploited to understand the contemporary epidemiology of common environmental pathogens. When used in conjunction with large epidemiological and survey type studies of exposure to microbes in water, soil, and food, the assay described here may provide valuable information to improve our understanding of the transmission of environmental pathogens. Furthermore, salivary antibody data obtained from our assay can be used to improve previously described dynamic and static risk assessment models (1, 5, 96, 97).

In conclusion, the multiplexed immunoassay presented here, together with the cutoff points established, allowed us to measure immunoprevalence rates and coinfections to six waterborne pathogens among beachgoers in Puerto Rico. A follow-up study will explore incident infections and attempt to elucidate the sources and health effects of exposure. Future work will exploit these techniques to examine exposure patterns in other communities. This assay, along with the approaches presented here, may enhance our knowledge and understanding of environmental microbial pathogenesis and assist risk assessment modelers.

## Ethics Statement

The IRB # 08-1844, University of North Carolina, Chapel Hill, NC, USA. Approval was obtained from the IRB # 08-1844, University of North Carolina, Chapel Hill, NC, USA, for the collection of saliva samples from beachgoers at Boquerón Beach, Puerto Rico, as part of the USEPA NEEAR Water Study. Study subjects provided informed consent and were instructed on the use of the Oracol™ saliva collection device. Infants younger than 1 year were not included. Informed consent was obtained from parents of minors.

## Author Contributions

SA designed the study with TW, AD, SG, GF, KO, and AG. TW, KO, AD, and ES provided the (NEEAR) saliva samples. SA, KS, SG, CC, and MS conducted the experiments. SA, TE, KS, SG, and LW were responsible for data analyses.

## Disclaimer

Mention of trade names or commercial products does not constitute endorsement or recommendation by the United States Environmental Protection Agency for use.

## Conflict of Interest Statement

The authors declare that the research was conducted in the absence of any commercial or financial relationships that could be construed as a potential conflict of interest.
